# Polish Adaptation of the Dutch Eating Behaviour Questionnaire (DEBQ): The Role of Eating Style in Explaining Food Intake—A Cross-Sectional Study

**DOI:** 10.3390/nu13124486

**Published:** 2021-12-15

**Authors:** Aleksandra Małachowska, Marzena Jeżewska-Zychowicz, Jerzy Gębski

**Affiliations:** Department of Food Market and Consumer Research, Institute of Human Nutrition Sciences, Warsaw University of Life Sciences (SGGW-WULS), Nowoursynowska 159C, 02-776 Warsaw, Poland; marzena_jezewska_zychowicz@sggw.edu.pl (M.J.-Z.); jerzy_gebski@sggw.edu.pl (J.G.)

**Keywords:** eating style, restrained eating, emotional eating, external eating, adaptation, DEBQ, food intake

## Abstract

Knowledge of associations between emotional, external, and restrained eating with food choices is still limited due to the inconsistent results of the previous research. The aim of the study was to adopt the Dutch Eating Behavior Questionnaire (DEBQ) and then to examine the relationship between emotional, external, and restrained eating styles and dietary patterns distinguished on the basis of intake of fruit and vegetables (fresh and processed separately), fruit and/or vegetable unsweetened juices, sweets and salty snacks, and the adequacy of fruit and vegetable intake. The cross-sectional study was conducted in 2020, in a sample of 1000 Polish adults. The questionnaire consisted of the Dutch Eating Behavior Questionnaire, questions on selected food groups intake, and metrics. DEBQ structure was tested using both exploratory and confirmatory factor analysis (EFA, CFA) and structural equation modelling (SEM), while multi-group analysis was used to test measurement invariance. Logistic regression was applied to investigate the association between eating styles and dietary patterns, identified with the use of K-means cluster analysis. EFA, CFA and SEM revealed a three-factor, 29-item tool with satisfactory psychometric parameters. Restrained eating (ResEat) and external eating (ExtEat) were found to decrease chances of low intake of both favorable (fruit, vegetables, and unsweetened juices) and unfavorable (sweets and salty snacks) foods and increased the chances of their moderate intake. ResEat increased the probability of the high intake of favorable and moderate or high intake of unfavorable foods. ResEat and ExtEat were predisposed to adequate intake of fruit and vegetables while emotional eating had the opposite effect. Gender, education, and BMI were also found to determine food intake. Our results provide evidence that both eating styles and sociodemographic characteristics should be taken into account while explaining food intake as they may favor healthy and unhealthy eating in different ways.

## 1. Introduction

Due to the complexity of dietary behaviors, diverse terms are used to describe their various aspects, i.e., dietary intake, diet, and eating habits [1]. Eating style (ES)—a construct involving some psychological factors such as food motives, feelings, and thoughts about food—is another term used to characterize dietary behaviors [2,3,4]. Eating style may determine inter alia susceptibility to weight changes, hence its evaluation might be useful in the assessment of one’s risk of overweight and obesity [5,6,7,8,9,10]. Amongst others ES, emotional, external, and restrained eating are distinguished in the literature [11].

Emotional eating (EmoEat) involves eating in response to emotions, both negative and positive, when food can be used as a coping mechanism for distress, sadness, and anxiety, or it might serve as a reward [5,12]. External eating (ExtEat), on the other hand, is a tendency to eat in response to environmental factors such as food availability, pleasant food aroma, or other people’s presence [13,14]. Restrained eating (ResEat) is mainly focused on restrictive dieting in order to regulate body weight [15]. There are several available scales to measure these constructs. For example, EmoEat can be assessed with the Emotional Eating Scale (EES) [16] or Emotional Overeating Questionnaire (EOQ) [17]. The Three-Factor Eating Questionnaire (TFEQ) [18], Revised Restraint Scale (RRS) [19], or Dieting and Weight History Questionnaire (DWHQ) [20] might be used to measure ResEat. Nonetheless, so far, there has only been one available tool devised to examine ExtEat—The Dutch Eating Behavior Questionnaire (DEBQ) [21]. Moreover, DEBQ is the only tool that can simultaneously measure all three constructs—EmoEat, ExtEat, and ResEat [22]. DEBQ validity has been previously tested in many populations [23,24,25,26,27,28,29,30]. To our knowledge, so far, DEBQ has not been adapted and validated in the representative sample of Polish population, yet its psychometric properties were confirmed for a sample with a preponderance of students [31]. Nevertheless, the possibility to use DEBQ in the Polish sample is limited.

Associations between eating styles were the focus of previous research but they still have not been unambiguously explained [11]. Restrained eating might be linked to the binge eating mechanism, thus increasing the risk of emotional and external eating [8,15]. Nonetheless, the relationship between ResEat, EmoEat, and ExtEat should be further studied due to the inconsistent results of available research [8,11,32,33]. It is known that restrained, emotional, and external eating might separately predispose one to greater calorie intake, especially from products high in fats and sugars, such as sweets, salty snacks, and fast-food [5,6,8,9,12,34,35,36,37]. Food intake is a result of complex interactions among eating behaviors; thus, modification in one of them may provoke changes in the others [38]. In previous studies, intake of individual food products was commonly assessed [5,6,13,34,36]. Thus, the results showing the relationship between eating style and food intake are still scarce as they relate to single ES and individual products, not to dietary patterns (DP).

The aim of the study was to adapt and evaluate psychometric parameters of the Dutch Eating Behavior Questionnaire in the representative sample of Polish adults aged 18–65. Moreover, the objective was to examine the relationship between emotional, external, and restrained eating styles, measured with DEBQ, and dietary patterns identified by the intake of selected food products as well as adequacy of fruit and vegetables intake. We hypothesize that different eating styles may diversely explain food intake, in a way that emotional and external eating would favor greater intake of unfavorable food products (sweets, salty snacks) and lower intake of favorable ones (fruit, vegetables), whereas restrained eating would be conducive to lower intake of unfavorable foods and greater intake of favorable ones. We believe that our study would contribute to a better understanding of eating styles and their relationships with food intake, including both healthy and unhealthy foods.

## 2. Materials and Methods

### 2.1. Study Design and Sample Collection

The study was conducted in February 2020 through a cross-sectional survey. A professional market research agency conducted recruitment from an e-panel consisting of approximately 60,000 registered individuals. Data were collected using the CAWI (Computer-Assisted Web Interview) technique. Gender, age, place of residence, and region were set as quota controls to ensure sample representativeness. The final sample consisted of 1000 participants aged 18–65 years who gave voluntary consent to participate in the study.

### 2.2. Instrument: The Dutch Eating Behavior Questionnaire (DEBQ)

The DEBQ [21] is an instrument developed to assess restrained, emotional, and external eating. Originally, it consisted of 33 items within 3 factors: 1/restrained eating (10 items, e.g., ‘How often do you try not to eat between meals because you are watching your weight?’), 2/emotional eating (13 items, e.g., ‘Do you get the desire to eat when you are anxious, worried or tense?‘), and 3/external eating (10 items; e.g., ‘If you see others eating, do you also have the desire to eat?’). The DEBQ uses a 5-point Likert scale, ranging from never (1) to very often (5). The average score is calculated for each subscale by adding scores obtained from individual items and dividing them by the number of items included in a subscale (mean range: 1–5). Higher scores in each subscale reflect higher level of restrained, emotional, and external eating, respectively.

The DEBQ was transculturally adapted by translating it into Polish by two independent translators. Then, jointly-agreed version was back-translated into English. Translation process continued until linguistic congruence between Polish and original version was achieved. The adapted scale was pretested in a group of 49 students, who reported no difficulties while completing the questionnaire.

### 2.3. Food Intake

Questions from the Dietary Habits and Nutrition Beliefs Questionnaire (KomPAN^®^) [39] were used to assess the frequency of intake of 5 food groups, i.e., fruit and vegetables (fresh and processed separately), fruit and/or vegetable unsweetened juices, sweets, and salty snacks. Participants rated frequency of eating on a 6-point scale ranging from never (1) to few times a day (6). Then, those categories were converted into values reflecting daily frequency of intake [40]. The amount consumed was assessed by asking participants how many portions of product they eat per day. Portion sizes were set as follows: 1 portion of vegetables and fruit (fresh and processed) equals 100 g, 1 portion of unsweetened juice equals 200 millilitres, and 1 portion of sweets and salty snacks equals 50 g. Each question was supplemented with exemplary portion sizes. Food intake was calculated for each food group by multiplying daily frequency of intake and amounts of portions consumed. Adequacy of total fruit and vegetables intake was assessed by summing fresh fruit, fresh and processed vegetables and unsweetened fruit, and vegetable or mixed juice intake in grams and comparing this value to recommendations established for Polish population—a minimum of 400 g of fruit and vegetables daily [41]. It was assumed that a maximum of 1 portion of unsweetened fruit, vegetables, or mixed juices (200 mL) can substitute for a maximum of 1 portion (100 g) of fruit or vegetables.

### 2.4. Statistical Analysis

Descriptive statistics were used to present sociodemographic characteristics and food intake in the study sample. The independence χ2 test was used to assess the diversity of socio-demographics between groups.

The factorial structure of the DEBQ was tested by running exploratory factor analysis (EFA) with varimax rotation with Kaiser normalization. The following criteria were selected to determine the final number of factors: eigenvalue ≥1.0, a scree plot test, interpretability of the solution, and factor loadings of at least 0.50. The factorability of the data was confirmed with the Kaiser–Meyer–Olkin (KMO) measure of sampling adequacy and Bartlett’s test of sphericity [42].

Confirmatory factor analysis (CFA) was performed to assess the fit of the factorial structure identified on the basis of EFA with the use of the following indices: the chi-square fit statistics/degree of freedom (Χ^2^/df), Tucker–Lewis index (TLI), comparative fix index (CFI), root mean square error of approximation (RMSEA), and root mean square residual (RMR). Good fit parameters included: Χ^2^/df below 2 or 3; TLI ≥ 0.95; CFI ≥ 0.95; RMSEA < 0.06; and the smallest RMR possible [43]. If the original model did not provide satisfactory fit parameters, item loading values as well as modification indices and standardized residual correlation values were used to modify the model [44].

The composite reliability (CR) indicating internal consistency was evaluated, given that values ≥ 0.70 are considered appropriate.

The Shapiro–Wilk test was applied to check the normality. Spearman’s correlation was used to measure a linear association between eating styles components.

Convergent validity was assessed by calculating average variance extracted (AVE), given that values should be equal to or greater than 0.50 [45]. Square root of AVE greater than the correlations between latent variables indicated established discriminant validity of the model [45].

Multi-group analysis was used to test measurement configural invariance (baseline, unconstrained model), metric invariance (factor loadings constrained to equality), and scalar invariance (factor loadings and item intercepts constrained to equality) across gender, age, and BMI status [46]. Based on the BMI-status, the sample was divided into 2 categories: 1/BMI < 25.0 kg/m^2^ and 2/BMI ≥ 25.0 kg/m^2^. According to the age, 4 groups were established: 18–24, 25–39, 40–54, and 55–65. Changes in the fit among models were assessed with the use of chi-square difference test. *p*-value greater than 0.05 indicated non-significant changes in the fit between models [24]. Partial measurement invariance testing was conducted if noninvariance was found by allowing problematic items to be freely estimated across groups [46].

Descriptive statistics were used to present scores for restrained, emotional, and external eating in total sample and subsamples. The Shapiro–Wilk test was applied to check the normality. Differences in scores among groups were examined with the use of Mann–Whitney test as well as One-Way ANOVA (Analysis of Variance) with post-hoc Waller–Duncan test. *p*-value lower than 0.05 was considered significant.

K-means cluster analysis was applied to identify groups, which were homogenous in terms of their food intake, i.e., fresh vegetables, processed vegetables, fresh fruit, unsweetened juices, sweets, and salty snacks [47]. A dendrogram was used to select the number of clusters, and the correctness of cluster separation was confirmed by CCC (cubic clustering criteria), the squared Euclidean distance, and Pseudo F statistics. One-Way ANOVA (Analysis of Variance) with post-hoc Waller–Duncan test was also performed to compare mean values of food intake and eating style components between clusters. *p*-value lower than 0.05 was considered significant.

Logistic regression analysis was applied to verify associations between mean scores on each subscale of DBEQ, gender, education, and body mass index (BMI), counted on self-reported weight and height (independent variables), and identified clusters separately (dependent variables in models 1–4) and the adequacy of fruit and vegetables intake (dependent variable in model 5). The variables describing eating styles (restrained, emotional, and external) and BMI were introduced into the models as continuous variables. Gender and education were treated as dichotomous variables with women and primary education being taken as references, respectively. Automatic selection of variables with the stepwise selection method was applied to the models. The parameters of the model (β) and point estimates eβ (OR) were estimated in the logistic regression models, with 95% confidence interval (CI). Significance was set at *p* < 0.05 for all analyses.

The analyses were performed using IBM SPSS Statistics for Windows, version 26.0 (IBM Corp, Armonk, NY, USA), AMOS graphics version 27.0, and SAS 9.4. Software (SAS Institute Inc., Cary, NC, USA).

## 3. Results

### 3.1. Characteristics of the Study Sample

Table 1 presents socio-demographic characteristics of participants. The total sample consisted of 1000 individuals (500 women and 500 men) aged 18–65 years. Mean age of participants was 41.3 years ( ± 13.6 standard deviation -SD).

### 3.2. Exploratory Factor Analysis (EFA)

EFA proved original three-factor structure of the DEBQ. The total variance explained was 64.51%. Out of all items, only item 31 was not included in the final model due to the factor loading being lower than 0.5 (Table 2). The rest of the items loaded into factors in the same way as in the original questionnaire; thus, the names of the factors remained unchanged.

### 3.3. Confirmatory Factor Analysis

The fit of the model identified during EFA with three correlated latent variables (emotional eating—13 items, restrained eating—10 items, and external eating—9 items) was examined (Appendix A, Table A1 Model a). Item 4 had low loading value (0.54) in comparison to other items (ranging from 0.79 to 1.04), hence it was eliminated (Model b). Then, modification indices were used to identify ‘error covariances’ and allow items within same factors to correlate (Items 8 and 9, r = 0.236; Items 11 and 16, r = 0.211; Items 12 and 14, r = 0.259; Items 12 and 20, r = −0.104; Items 12 and 23, r = 0.429; Items 13 and 14, r = 0.087; Items 14 and 18, r = −0.137; Items 14 and 20, r = −0.115; Items 14 and 23, r = 0.159; Items 15 and 21, r = 0.148; Items 16 and 22 = −0.154; Items 18 and 20, r = 0.091; Items 24 and 25, r = 0.266; Items 24 and 27, r = 0.155; Items 24 and 30, r = −0.057; Items 25 and 26, r = 0.143; Items 26 and 31, r = −0.209; Items 28 and 29, r = 0.247; Items 30 and 31, r = 0.267; and Model c). Items 26 and 32 were further removed due to high standardized residual covariance values (> 4) with multiple items (Model d). The final model (d) proved satisfactory fit indices.

Psychometric parameters, including discriminant validity, convergent validity, and composite reliability, were tested. The final 29-item model demonstrated acceptable parameters (Table 3).

Relationships among eating styles were examined. Moderate correlations were found between ExtEat and EmoEat (r = 0.49), while other relationships were weak (Table 4).

### 3.4. Multiple-Group Analysis

Measurement invariance across gender, BMI-status, and age groups was tested by comparing fit measures of created models (Table 5). Metric and scalar invariance was supported across gender (*p* > 0.05). While examining models across groups according to BMI-status, metric noninvariance was found. The constraints for sources of noninvariance (Item 2, *p* = 0.030; Item 5, *p* = 0.012) were released and model was retested, resulting in partial metric invariance (Adj. Model 1). The scalar invariance (Model 2) across BMI-status was achieved. Metric invariance was established across groups according to age (Model 1); however, scalar invariance was not confirmed, which required further testing. Three items were found to significantly decrease model fit (Item 9, *p* = 0.001; Item 27, *p* = 0.003; and Item 29, *p* = 0.003); thus, they were allowed to be freely estimated by releasing the constraints across age groups. Retesting the model proved acceptable results, hence partial scalar invariance was obtained (Adj. Model 2).

Table 6 presents mean scores for restrained, emotional, and external eating in subsamples. Gender was found to differentiate only level of restrained eating in a way that women had significantly higher level than men. Similarly, only restrained eating was found to significantly differ in groups according to BMI-status. Participants with greater BMI (≥ 25 kg/m^2^) displayed greater intensity of restrained eating in comparison to respondents with BMI lower than 25 kg/m^2^. People aged 55–65 had the lowest level of both emotional and external eating, whereas among those aged 18–24, the highest level of those eating styles were observed. Restrained eating did not significantly differ across age groups.

### 3.5. Food Intake

Frequency and quantity of intake of selected foods are presented in Table 7. More participants consumed fresh vegetables and fruit at least once a day than processed ones (32.4 and 42.6 vs. 17.4 and 7.9%, respectively). Moreover, more respondents ate sweets (20.1%) compared to salty snacks (6.8%) at least once a day. Fresh fruit were the most consumed product (2.1 ± 2.4 portions per day), whereas salty snacks were the least consumed product (0.5 ± 1.2 portions per day).

The food intake varied between clusters (Table 8). Cluster 1 (‘Low intake’) was characterized by the lowest intake of all food groups. No significant differences were found between cluster 1 and 2 in the intake of sweets and salty snacks. Cluster 2 was named as ‘Moderate intake’ because intake of each food group was higher than in cluster 1 and lower than in other clusters, except for the intake of sweets and salty snacks in cluster 4. There were no differences in the amount of fresh fruit and processed vegetables consumed in clusters 3 and 4. Despite the differences observed between the intake of unsweetened juices and fresh vegetables between two clusters, intake of these foods was considered high in both of them. The highest intake of salty snacks was observed in cluster 3 (“High intake’).

### 3.6. Eating Styles and Their Relationship with Food Intake

Table 9 illustrates mean values of eating styles in the study sample and among clusters. The ‘Low intake’ cluster was characterized by the lowest mean scores for restrained eating (ResEat) and external eating (ExtEat). In cluster 3, the highest score for ExtEat was observed, while in cluster 4, the highest restrained eating score was recorded. The scores on emotional eating did not differentiate the clusters.

### 3.7. Relationship between Eating Styles and Food Intake

Relationships among eating styles, gender, education, BMI, and adherence to each cluster are presented in Table 10. The chance of adherence to cluster ‘Low intake’ decreased with higher score on restrained (ResEat) and external (ExtEat) eating. Each subsequent point on these subscales decreased adherence to that cluster by 24% and 21%, respectively. Men were more likely to adhere to ‘Low intake’ cluster compared to women (OR: 1.92; 95% CI: 1.47–2.51). Chances to adhere to this cluster decreased with higher level of education. People with lower secondary education were less likely to adhere to cluster 1 by 39%, those with upper secondary education by 54%, and those with higher education by 59%, compared to people with primary education.

The chance of adherence to cluster ‘Moderate intake’ increased with higher score on restrained eating and external eating. Each subsequent point on ResEat and ExtEat increased the adherence to this cluster by 21% and 28%, respectively. Men were less likely to adhere to “Moderate intake” cluster compared to women (OR: 0.71; 95% CI: 0.53–0.94).

The chance of adherence to cluster ‘High intake’ increased with higher score on restrained eating. Each subsequent point on ResEat increased the adherence to this cluster ‘by 64% (OR: 1.64; 95% CI: 1.01–2.67). Similarly, each subsequent point on ResEat increased the adherence to cluster ‘High intake of FV and moderate intake of SS’ by 64% (OR: 1.64; 95% CI: 1.22–2.20). Moreover, people with higher education were more likely to adhere to this cluster by 639% compared to people with primary education, while those with upper secondary education and lower secondary education by 386% and 192%, respectively. Men were less likely to adhere to ‘High intake of FV and moderate intake of SS’ cluster compared to women (OR: 0.30; 95% CI: 0.16–0.56). Moreover, the chance of adherence to this cluster decreased with higher BMI (OR: 0.93; 95% CI: 0.87–0.99).

Relationships among eating styles and adequacy of fruit and vegetables intake (F&V) are presented in Table 11.

The chance of adequate intake of F&V increased with higher score on restrained eating and external eating. Each subsequent point of these scales increased the adequate intake by 56% (OR: 1.56; 95% CI: 1.33–1.83), and by 43% (OR: 1.43; 95% CI: 1.16–1.76), respectively. The chance of adequate F&V intake decreased with each subsequent point on emotional eating by 20% (OR: 0.80; 95% CI: 0.68–0.95). Men were less likely to have an adequate intake of F&V compared to women (OR: 0.45; 95% CI: 0.35–0.59). People with higher education were more likely to have an adequate F&V intake by 161% compared to people with primary education, and those with upper secondary education and lower secondary education by 120% and 73%, respectively.

## 4. Discussion

We aimed to investigate dimensional structure of the Dutch Eating Behavior Questionnaire in the representative sample of Polish adults using both exploratory and confirmatory factor analysis as well as structural equation modelling. Despite including reverse scoring of item 31 proposed by van Strien et al. [21], this item was eliminated from further testing due to low factor loading, which was also noted by other researchers [23,28]. Decision on further elimination of items (Items 4, 26, and 32) was made to improve fit indices of the final model. A previous study on the adaptation of DEBQ in Polish population confirmed original, three-factorial structure with 33 items. However, study group involved participants aged 18–37 [31]. In the current study, wider age range group (18–65 years) was selected with the use of quotas to ensure a representative sample. Differences in the sample collection as well as different methods used to assess models fit may explain why our results varied from those published by Malesza [31]. We have noted partial invariance across BMI-status and age groups and full invariance across gender. Noninvariance of two loadings across BMI groups suggests that they are more closely related to factor in one subgroup than in other, whereas noninvariance of three item intercepts across age groups means that behaviors described within these particular items are more prevalent among some age groups; however, this increased commonness is not linked to increased intensity of factors [46]. So far, there is lack of consensus on the most adequate way to assess measurement invariance and differences among models [46]. Various methods used by the authors of previous research on DEBQ adaptation [24,25,26] might cause lower or higher levels of achieved invariances [46].

The decision on including fruit, vegetables, unsweetened juices, sweets, and salty snacks to identify clusters was motivated mostly by their inadequate intake in Poland and in other countries, as well as due to global dietary guidelines encouraging greater intake of fruit and vegetables and limited salt and sugar intake from highly-processed foods such as sweets and salty snacks [41,48]. EmoEat did not predict adherence to identified clusters but it lowered the chances of adequate intake of fruit and vegetables. Available studies suggest that EmoEat predisposes one to higher intake of highly caloric foods, such as sweets and salty snacks [5,6,36], which may possibly lead to lower intake of healthier foods, such as fruit and vegetables. However, our results do not support this hypothesis. This may be caused by the fact that in the DEBQ, EmoEat subscale focuses on increased intake while experiencing negative emotions, such as disappointment, boredom, or loneliness [21]. However, both negative and positive emotions may have an impact on food choices [12,49]. Thus, future studies should include both positive and negative emotions while attempting to explain how EmoEat affects food intake.

External eating (ExtEat) decreased chances of adhering to ‘Low intake’ cluster and increased chances of adherence to ‘Moderate intake’ one. ExtEat also increased chances of adequate intake of fruit and vegetables. On the other hand, the highest ExtEat score was observed in cluster ‘High intake’, which is consistent with externality theory [50]. However, few previous studies questioned the role of ExtEat in overweight and obesity [37,51,52]. Our results show that ExtEat did not vary among different BMI groups, and it also increased the likelihood of adequate fruit and vegetables intake, which is in line with the findings that ExtEat might not be a strong predictor of weight gain [51,52]. Additionally, some external stimuli, i.e., positive social modelling, may favor moderate intake (cluster 2) [53]. Our findings regarding ExtEat should be verified in future research with the use of additional questions, for example, concerning the frequency of eating in response to external cues [37].

Only restrained eating (ResEat) explained adherence to clusters characterized by the greatest intake of fruit and vegetables and moderate intake of sweets and salty snacks, which most closely reflects dietary recommendations [48]. ResEat was also predisposed to adequate intake of fruit and vegetables. Nevertheless, promoting restrained eating to improve one’s dietary habits in the long-term is controversial due to the results showing correlation between ResEat intensity, poorer psychological parameters, higher risk of binge eating episodes, or even higher BMI [9,10,15,35,54]. Those aspects were not included in the study, except for BMI. BMI lowered chances of adherence to the ‘High intake of F&V and moderate intake of SS’ cluster [55,56], thus revealing opposite effect to ResEat. Dietary restraints may affect food intake differently among underweight, normal-weight, overweight, and obese individuals, suggesting that this parameter should be included in the studies on ResEat construct [54,57]. In our study, ResEat also lowered the chances of low intake of fruit, vegetables, unsweetened juices, sweets, and salty snacks (cluster 1) and increased the chances of moderate intake of them (cluster 2), which provides additional evidence that applying restrictions may result in lower energy intake [9,36,58]. Nonetheless, ResEat may also favor greater calorie intake [8,35]. We have noted that ResEat increased chances of high intake of both favorable and unfavorable foods (cluster 3 ‘High intake’). In ‘restrained eating theory’ and ‘boundary model’, ResEat may lead to dysregulation of eating, which might explain higher intake of foods as a result of restrictions [59,60]. Further studies on the relationship between ResEat and food intake should include other variables as mediators of this correlation (i.e., psychological health parameters, such as depressive symptoms [5,36], nutritional knowledge [61], frequency of dieting and diet duration [8], and/or physical activity [10]), as well as other foods than those included in our study. Moreover, the use of different tools than DEBQ to determine this association is also desirable due to the complexity of ResEat construct [62].

Links between restrained, emotional, and external eating [8,15] were confirmed by positive correlations in our study, yet their strength was mostly weak. Only EmoEat and ExtEat were found to moderately correlate (r = 0.49). Since previous studies on relationship between eating styles have shown incoherent results [8,11,32,33], future research is necessary.

Sociodemographic characteristics, i.e., gender and education level, also showed a predictive effect in the logistic regression models. Men were more likely to adhere to ‘Low intake’ and less likely to adhere to the ‘Moderate intake’ and ‘High intake’ clusters. These findings may seem inconsistent with previous results confirming higher food intake in men than women. However, men consume other food such as meat and meat products or eggs in the greater quantity than women [63,64,65], and these products were not included to the analysis. The chances for men to comply with dietary recommendations for fruit and vegetables were 55% lower compared to women, as was observed in other studies [64,65]. It can be assumed that higher chance of men being in the ‘Low intake’ cluster along with the decrease in the scores for ResEat may explain the failure to meet the recommendations for fruit and vegetables in men [36,66,67]. Few available studies have shown that females have higher levels of ResEat and EmoEat than males, and no differences can be found for ExtEat [9,24,25,26]. Our findings partially confirm those observations as no differences were found for EmoEat across gender groups. Some studies on DEBQ adaptation did not provide data on eating styles levels in men and women separately, including previous study in the Polish population [23,28,31]. Moreover, due to the fact that previous studies on eating styles and their association with food intake were often conducted in homogenous groups, including women [8,11,12,13,30,53,58], it is suggested to continue them in both men and women from different age groups to confirm our findings. Higher education increased chances of adherence to the most favorable cluster ’High intake of FV and moderate intake of SS’) and decreased chances of adherence to the least favorable ‘Low intake’ cluster, which is coherent with previous studies [68,69,70]. Nevertheless, more research on the assessment of food intake with the inclusion of all food groups is needed with particular attention paid to education level among females and males separately to refer to large number of men in the ‘Low intake’ cluster and clarify the role of education in explaining food intake among men.

### Strengths and Limitations

A relatively large, representative study group, which enables generalization of the results in Polish population, is a strength of the study. Moreover, to our knowledge, our study is the first to measure restrained, emotional, and external eating in people aged 18–65, both men and women, and their relationship with food intake in Polish population. However, some limitations should be considered. Due to the cross-sectional design of the study, the causality of identified relationships among variables cannot be determined. As the study was conducted solely among Polish adults, the findings cannot be generalized in populations with different cultural backgrounds. Despite the fact that analyzed psychometric parameters of the final model were satisfactory, the external validity of the 29-item DEBQ was not tested, which may be pointed as another study limitation. Moreover, food intake was assessed on the basis of dietary patterns distinguished only on the basis of selected food groups intake, i.e., fruit, vegetables, unsweetened juices, sweets, and salty snacks. Additionally, food intake might have been underreported by the participants and hence have affected results of further analysis, including identified clusters. However, according to data provided by Statistics Poland in a report called ‘Household’s Budget Survey in 2020′, medium monthly consumption per capita of food products analyzed in the current study, i.e., fruit, vegetables, juices, and sweets, seems to be rather low, which is in line with our findings [71]. Sparse effect that may increase the probability of monotone likelihood is a further limitation [72].

## 5. Conclusions

We managed to confirm a three-factor structure of the Dutch Eating Behavior Questionnaire in the Polish population. Identification of items, which significantly decreased model fit parameters, allowed one to obtain a 29-item tool with satisfactory psychometric parameters, including full measurement invariance (across gender) and partial measurement invariance (across groups identified based on BMI-status and age). The findings proved that some eating styles may explain healthy and unhealthy food intake. External eating was found to decrease chances of low intake of both favorable (fruit, vegetables, and unsweetened juices) and unfavorable foods (sweets, salty snacks). At the same, it predisposed one to moderate intake of all analyzed products. Findings regarding restrained eating have shown that this style may increase both moderate and high intake of fruit, vegetables, sweets, and salty snacks and, simultaneously, lower the chances of low intake of all of these products. Restrained eating and external eating were conducive to higher chances of adequate intake of fruit and vegetables, while emotional eating lowered these chances. Due to our observations on complex correlations between eating styles and food choices, it seems that future studies should search for possible mediators of these relationships to obtain a better understanding of the way eating styles can determine food intake. Despite a weak correlation between restrained and external eating, they were found to similarly predict food intake, which should be confirmed in further studies, including testing for a mediating role of external eating on the association between restrained eating and food intake. Higher education and female gender were conducive to high intake of fruit, vegetables, and unsweetened juices and to moderate intake of sweets and salty snacks, whereas greater BMI lowered the chances of such intake. Our results provide evidence that both eating styles as well as sociodemographic characteristics should be taken into account while explaining food intake, as they may favor healthy and unhealthy eating in different ways.

## Figures and Tables

**Table 1:** Socio-demographic characteristics of the study sample.

Variables		Total (N = 1000) N (%)	Clusters			
			1 ‘Low Intake’ (N = 624)	2 ‘Moderate Intake’ (N = 281)	3 ‘High Intake’ (N = 27)	4 ‘High Intake of FV and Moderate Intake of SS’(N = 68)
Gender ***	Women	500 (50.0)	274 (43.9)	159 (56.6)	14 (51.8)	53 (77.9)
	Men	500 (50.0)	350 (56.1)	122 (43.4)	13 (48.2)	15 (22.1)
Age [in years]	18–24	112 (11.2)	82 (13.1)	24 (8.5)	1 (3.7)	5 (7.4)
	25–39	351 (35.1)	206 (33.1)	105 (37.4)	14 (51.9)	26 (38.2)
	40–54	304 (30.4)	193 (30.9)	82 (29.2)	9 (33.3)	20 (29.4)
	55–65	233 (23.3)	143 (22.9)	70 (24.9)	3 (11.1)	17 (25.0)
Education ***	Primary	171 (17.1)	129 (20.7)	35 (12.5)	4 (14.8)	3 (4.4)
	Lower secondary	240 (24.0)	157 (25.2)	67 (23.8)	5 (18.5)	11 (16.2)
	Upper secondary	343 (34.3)	203 (32.5)	103 (36.6)	12 (44.4)	25 (36.8)
	Higher (e.g., BSc, Msc)	246 (24.6)	135 (21.6)	76 (27.1)	6 (22.2)	29 (42.6)
Place of Residence	Village	373 (37.3)	231 (37.0)	110 (39.2)	10 (37.1)	22 (32.2)
	Town below 20,000 inhabitants	31 (13.1)	93 (14.9)	29 (10.3)	3 (11.1)	6 (8.8)
	Town between 20,000 and 100,000 inhabitants	183 (18.3)	116 (18.6)	51 (18.2)	5 (18.5)	11 (16.3)
	City over 100,000 inhabitants	313 (31.3)	184 (29.5)	91 (32.3)	9 (33.3)	29 (42.7)

N—number of participants, FV—fresh vegetables, processed vegetables, fresh fruit, and unsweetened juices); SS—sweets and salty snacks; and *** significant differences between clusters at *p* < 0.001 (the independence χ^2^).

**Table 2:** Component loadings for DEBQ items.

DEBQ Items	Emotional Eating	Restrained Eating	External Eating
Item 1 ^a^	0.191	0.795 *	0.091
Item 2	0.116	0.720 *	0.072
Item 3	0.203	0.817 *	0.009
Item 4	−0.190	0.576 *	0.215
Item 5	0.324	0.692 *	−0.014
Item 6	0.247	0.743 *	0.070
Item 7	0.169	0.810 *	0.042
Item 8	0.017	0.766 *	0.049
Item 9	0.042	0.767 *	−0.007
Item 10	0.168	0.783 *	0.038
Item 11	0.858*	0.149	0.165
Item 12	0.663*	0.059	0.378
Item 13	0.849*	0.113	0.234
Item 14	0.813 *	0.093	0.242
Item 15	0.860 *	0.135	0.175
Item 16	0.858 *	0.143	0.172
Item 17	0.867 *	0.144	0.175
Item 18	0.854 *	0.152	0.192
Item 19	0.858 *	0.129	0.194
Item 20	0.876 *	0.168	0.138
Item 21	0.858 *	0.124	0.224
Item 22	0.860 *	0.120	0.181
Item 23	0.764 *	0.077	0.316
Item 24	0.174	0.012	0.781 *
Item 25	0.220	0.041	0.772 *
Item 26	0.093	0.085	0.780 *
Item 27	0.172	0.044	0.688 *
Item 28	0.225	0.106	0.715 *
Item 29	0.277	0.087	0.705 *
Item 30	0.344	0.108	0.655 *
Item 31	0.001	−0.313	−0.190
Item 32	0.472	0.109	0.552 *
Item 33	0.190	0.104	0.672 *
*Eigenvalue % of Variance*	10.051	5.999	5.237
	30.457	18.180	15.868

^a^ original DEBQ number of the statement (1–10 restrained eating, 11–23 emotional eating, and 24–33 external eating); * loadings > 0.50.

**Table 3:** Validity and reliability of the final DEBQ model.

Factors	Parameters		
	AVE	Square Root of AVE	CR
Restrained eating (9 items)	0.57	0.76	0.92
Emotional eating (13 items)	0.74	0.86	0.97
External eating (7 items)	0.50	0.70	0.87

AVE—average variance extracted, CR—composite reliability.

**Table 4:** Correlations between eating style subscales.

Variables	1	2	3
1. ResEat	-		
2. EmoEat	0.31 **	-	
3. ExtEat	0.16 **	0.49 **	-

ResEat—restrained eating; EmoEat—emotional eating; ExtEat—external eating; ** Significant at *p* < 0.01 (Spearman’s correlation).

**Table 5:** Fit measures for the DEBQ multi-group models.

Models	Fit measures					Model Comparison			
	Χ^2^	df	Χ^2^/df	RMSEA	CFI	Comparison	Δ Χ^2^	Δdf	*p*
Gender									
Model 0 *	1532.997	716	2.141	0.034	0.967	-	-	-	-
Model 1 **	1550.432	742	2.090	0.033	0.967	Model 1 vs. 0	17.435	26	0.895
Model 2 ***	1581.765	768	2.060	0.033	0.967	Model 2 vs. 1	31.333	26	0.216
BMI status									
Model 0 *	1496.383	716	2.090	0.033	0.968	-	-	-	-
Model 1 **	1537.823	742	2.073	0.033	0.967	Model 1 vs. 0	41.440	26	00.028
Adj. Model 1 **	1532.015	740	2.070	0.033	0.967	Adj. Model 1 vs. 0	35.632	24	0.060
Model 2 ***	1558.506	764	2.040	0.032	0.967	Model 2 vs. Adj. Model 1	26.491	24	0.329
Age									
Model 0 *	2495.885	1432	1.743	0.027	0.957	-	-	-	-
Model 1 **	2571.669	1510	1.703	0.027	0.957	Model 1 vs. 0	75.784	78	0.550
Model 2 ***	2691.160	1588	1.695	0.027	0.956	Model 2 vs. 1	119.491	78	0.002
Adj. Model 2 ***	2649.722	1579	1.678	0.026	0.957	Adj. Model 1 vs. Model 1	78.053	69	0.213

*p*—significance value; Χ^2^/df—chi-square fit statistics/degree of freedom; RMSEA—root mean square error of approximation; and adj.—adjusted. * Model 0—configural model (unconstrained); ** Model 1—metric model; adj.—adjusted; and *** Model 2—scalar model.

**Table 6:** Mean scores for emotional, restrained, and external eating.

Groups	Restrained Eating	Emotional Eating	External Eating
Total sample Gender *	2.81 ± 0.92	2.30 ± 1.00	2.98 ± 0.77
Women (*n* = 500)	2.89 ± 0.91 ^b^	2.31 ± 0.98	2.99 ± 0.77
Men (*n* = 500)	2.74 ± 0.92 ^a^	2.28 ± 1.01	2.97 ± 0.77
BMI-status **			
<25 kg/m2 (*n* = 522)	2.68 ± 0.97 ^b^	2.31 ± 1.01	2.99 ± 0.79
≥25 kg/m2 (*n* = 478)	2.96 ± 0.84 ^a^	2.28 ± 0.98	2.97 ± 0.74
Age [in years] **			
18–24 (*n* = 112)	2.70 ± 0.90	2.56 ± 0.91 ^c^	3.16 ± 0.74 ^c^
25–39 (*n* = 351)	2.75 ± 0.96	2.45 ± 1.08 ^c^	3.06 ± 0.83 ^bc^
40–54 (*n* = 304)	2.88 ± 0.86	2.25 ± 0.95 ^b^	2.95 ± 0.75 ^b^
55–65 (*n* = 233)	2.86 ± 0.92	1.99 ± 0.87 ^a^	2.80 ± 0.67 ^a^

Significant at * *p* < 0.05, ** *p* < 0.001; Mann–Whitney U Test, One-Way Analysis of Variance with Waller–Duncan post hoc test; and a,b,c—means with different letters are significantly different among groups.

**Table 7:** Food intake in the study sample.

Variables	Frequency of Intake (%)		Number of Portions a Day (%)		Daily Intake in Portions (incl. frequency)	Daily Intake (g/mL)
	Less than Once a Day	At Least Once a Day	Less than One Portion a Day	At Least One Portion a Day	M ± SD	M ± SD
**Fresh vegetables**	67.6	32.4	17.7	82.3	1.6 ± 2.2	162.5 ± 220.0
**Processed vegetables**	82.6	17.4	12.8	87.2	1.0 ± 1.5	101.8 ± 145.9
**Fresh fruit**	57.4	42.6	9.1	90.9	2.1 ± 2.4	205.5 ± 240.9
**Processed fruit**	92.1	7.9	33.0	67.0	0.6 ± 1.2	nd
**Unsweetened juices**	88.4	11.6	29.9	70.1	0.7 ± 1.6	144.7 ± 313.7
**Sweets**	79.9	20.1	20.3	79.7	1.0 ± 1.7	51.0 ± 83.5
**Salty snacks**	93.2	6.8	37.5	62.5	0.5 ± 1.2	26.3 ± 57.3

N—number of participants; M—mean; SD—standard deviation; and nd—no data.

**Table 8:** Clusters’ profile according to the amount of food consumed.

Food Groups	Food Intake Mean ±SD (N = 1000)	Clusters				
		1 ‘Low Intake’ (N = 624)	2 ‘Moderate Intake’ (N = 281)	3 ‘High Intake’ (N = 27)	4 ‘High Intake of FV and Moderate Intake of SS’ (N = 68)	*p*-Value
Fresh vegetables	162.5 ± 275.7	60.6 ^d^ ± 69.76	231.9 ^c^ ± 167.7	460.0 ^b^ ± 347.3	691.91 ^a^ ± 275.7	<0.0001
Processed vegetables	101.8 ± 248.5	51.2 ^c^ ± 54.31	141.4 ^b^ ± 138.3	315.2 ^a^ ± 370.1	316.1 ^a^ ± 248.5	<0.0001
Fresh fruit	205.5 ± 264.7	78.1 ^c^ ± 69.12	343.6 ^b^ ± 210.1	614.0 ^a^ ± 440.4	642.0 ^a^ ± 264.7	<0.0001
Unsweetened juices	144.7 ± 251.1	44.6 ^d^ ± 74.65	181.3 ^c^ ± 193.9	1667.0 ^a^ ± 594.8	307.9 ^b^ ± 251.1	<0.0001
Sweets	51.0 ± 112.9	38.4 ^c^ ± 65.90	62.5 ^bc^ ± 88.40	148.2 ^a^ ± 171.9	80.3 ^b^ ± 112.9	<0.0001
Salty snacks	26.3 ± 72.73	19.3 ^c^ ± 41.84	32.1 ^bc^ ± 62.98	91.7 ^a^ ± 146.3	39.2 ^b^ ± 72.73	<0.0001

FV—fresh vegetables, processed vegetables, fresh fruit, and unsweetened juices; SS—sweets and salty snacks; One-Way ANOVA (Analysis of Variance); and a,b,c,d,e—means with the same letter are not significantly different in Waller–Duncan test.

**Table 9:** Clusters’ profiles according to eating style subscales in the study sample.

Eating Styles	Total (N = 1000)	Clusters (M ± SD)			
		1 ‘Low Intake’ (N = 624)	2 ‘Moderate Intake’ (N = 281)	3 ‘High Intake’ (N = 27)	4 ‘High Intake of FV and Moderate Intake of SS’ (N = 68)
Restrained eating (ResEat) *	2.81 ± 0.92	2.70 ^ab^ ± 0.91	2.96 ^ac^ ± 0.86	2.82 ± 1.21	3.21 ^bc^ ± 0.88
Emotional eating (EmoEat)	2.30 ± 1.00	2.26 ± 0.96	2.35 ± 1.04	2.41 ± 1.21	2.30 ± 1.02
External eating (ExtEat) **	2.98 ± 0.77	2.91 ^ab^ ± 0.65	3.10 ^a^ ± 0.75	3.27 ^b^ ± 0.92	2.95 ± 0.78

N—number of participants; M—mean; SD—standard deviation; * significant at *p* < 0.001; ** significant at *p* = 0.001; One-Way ANOVA (Analysis of Variance); and a,b,c^,^—means with the same letter are significantly different in Waller–Duncan test.

**Table 10:** Cluster’s odds ratios (OR; 95% CI) in the study sample.

Parameter	Level of variable	β	OR	95% Cl		*p*
**Model 1—adherence to Cluster 1 ‘Low intake’**						
Intercept		2.284				<0.0001
Restrained eating		−0.271	0.76	0.66	0.89	0.0004
External eating		−0.236	0.79	0.66	0.94	0.0095
Gender	Men	0.655	1.92	1.47	2.51	<0.0001
	Women (ref.)	0	1	-	-	-
Education	Lower secondary	−0.487	0.61	0.39	0.96	0.0332
	Upper secondary	−0.770	0.46	0.30	0.70	0.0003
	Higher	−0.881	0.41	0.27	0.64	<0.0001
	Primary (ref.)	0	1	-	-	-
**Model 2—adherence to Cluster 2 ‘Moderate intake’**						
Intercept		−2.059				<0.0001
Restrained eating		0.189	1.21	1.03	1.41	0.0180
External eating		0.247	1.28	1.06	1.54	0.0094
Gender	Men	−0.345	0.71	0.53	0.94	0.0159
	Women (ref.)	0	1	-	-	-
**Model 3—adherence to Cluster 3 ‘High intake’**						
Intercept		−5.132				<0.0001
Restrained eating		0.496	1.64	1.01	2.67	0.0455
**Model 4—adherence to Cluster 4 ‘High intake of FV and moderate intake of SS’**						
Intercept		−3.419				0.0004
Restrained eating (ResEat)		0.495	1.64	1.22	2.20	0.0009
Gender	Men	−1.190	0.30	0.16	0.56	0.0001
	Women (ref.)	0	1	-	-	-
BMI [in kg/m^2^]		−0.069	0.93	0.88	0.99	0.0315
Education	Lower secondary	1.072	2.92	0.79	10.81	0.1081
	Upper secondary	1.580	4.86	1.42	16.55	0.0116
	Higher	2.001	7.39	2.18	25.07	0.0013
	Primary (ref.)	0	1	-	-	-

Ref.—reference group; OR—point estimate (eβ); (95% CI); and 95% Wald Confidence.

**Table 11:** Adequate intake of fruit and vegetables’ odds ratios (OR; 95% CI) in the study sample.

Parameter	Level of Variable	β	OR	95% Cl		*p*
Intercept		−2.324				<0.0001
Restrained eating		0.444	1.56	1.33	1.83	<0.0001
Emotional eating		−0.220	0.80	0.68	0.95	0.0088
External eating		0.356	1.43	1.16	1.76	0.0008
Gender	Men	−0.791	0.45	0.35	0.59	<0.0001
	Women (ref.)	0	1	-	-	-
Education	Lower secondary	0.550	1.73	1.12	2.68	0.0136
	Upper secondary	0.789	2.20	1.46	3.32	0.0002
	Higher	0.959	2.61	1.69	4.03	<0.0001
	Primary (ref.)	0	1	-	-	-

Ref.—reference group; OR—point estimate (eβ); (95% CI); and 95% Wald Confidence.

## Data Availability

The data presented in this study are available on request from the corresponding author.

## Appendix A

**Table A1:** Fit measures for the DEBQ models.

Models	Fit Indices					
	*p*	Χ^2^/df	TLI	CFI	RMSEA	RMR
Model a	<0.001	4.469	0.934	0.938	0.059	0.077
Model b	<0.001	4.376	0.939	0.943	0.058	0.071
Model c	<0.001	2.750	0.968	0.972	0.042	0.067
Model d	<0.001	2.519	0.974	0.977	0.039	0.057

*p*—significance value, Χ^2^/df—chi-square fit statistics/degree of freedom, TLI—Tucker–Lewis index, CFI—comparative fix index, RMSEA—root mean square error of approximation, and RMR—root mean square residual.
